# MIFNN: Molecular Information Feature Extraction and Fusion Deep Neural Network for Screening Potential Drugs

**DOI:** 10.3390/cimb44110382

**Published:** 2022-11-13

**Authors:** Jingjing Wang, Hongzhen Li, Wenhan Zhao, Tinglin Pang, Zengzhao Sun, Bo Zhang, Huaqiang Xu

**Affiliations:** School of Physics and Electronic Science, Shandong Normal University, No.1, University Road, Science Park, Changqing District, Jinan 250358, China

**Keywords:** feature fusion, deep learning, particle swarm optimization, attention mechanism, support vector machine

## Abstract

Molecular property prediction is essential for drug screening and reducing the cost of drug discovery. Current approaches combined with deep learning for drug prediction have proven their viability. Based on the previous deep learning networks, we propose the Molecular Information Fusion Neural Network (MIFNN). The features of MIFNN are as follows: (1) we extracted directed molecular information using 1D-CNN and the Morgan fingerprint using 2D-CNN to obtain more comprehensive feature information; (2) we fused two molecular features from one-dimensional and two-dimensional space, and we used the directed message-passing method to reduce the repeated collection of information and improve efficiency; (3) we used a bidirectional long short-term memory and attention module to adjust the molecular feature information and improve classification accuracy; (4) we used the particle swarm optimization algorithm to improve the traditional support vector machine. We tested the performance of the model on eight publicly available datasets. In addition to comparing the overall classification capability with the baseline model, we conducted a series of ablation experiments to verify the optimization of different modules in the model. Compared with the baseline model, our model achieved a maximum improvement of 14% on the ToxCast dataset. The performance was very stable on most datasets. On the basis of the current experimental results, MIFNN performed better than previous models on the datasets applied in this paper.

## 1. Introduction

The discovery of new drugs or the reuse of drugs is a popular task in biochemistry. Prediction of drug effectiveness or toxicity based on molecular properties plays an important role in this task. In recent years, with the development of machine learning, especially the emergence of deep learning [1,2], many methods have achieved better performance in this task. Drug molecules or compounds are converted into computer-recognizable formats, such as molecular maps [3], molecular fingerprints [4], and molecular descriptors. These readable forms of molecular information are extracted by various means, including deep learning, to form unique molecular features that can be used to achieve subsequent classification or prediction tasks.

Schneider’s studies [5] have shown that, if different kinds of molecular information can more comprehensively contain molecular biochemical information after feature extraction, this will directly improve prediction accuracy. Molecular descriptors and fingerprints are usually designed for various specific chemical and biological tasks. For example, one of the most classical molecular descriptors is the Simplified Input Line Entry System (SMILES) for retrieving molecular information. Molecular descriptors are designed for special tasks related to molecules. They are more flexible than molecular fingerprints and have their advantages. SMILES is especially used for molecular retrieval [6], and it has been widely used in molecular datasets. The datasets used in this paper also use SMILES as a general retrieval format. Yang et al. [7] proposed molecular directed information. The team also proposed a Chemprop model based on the molecular descriptor, which has achieved great success in molecular screening. On the basis of the success of molecular directed information in the field of molecular screening, this paper selects it as part of the input sequence.

Later, Durant et al. [8] proposed a key-based molecular fingerprint, Molecular Access System (MACCS), to retrieve molecules by molecular substructure. With the development of deep learning and various complex needs, some studies began to build molecular descriptors and molecular fingerprints on the basis of the spatial coordinate information of molecules at the three-dimensional level, such as the 3Dmol network proposed by Chunyan Li et al. [9] and the 4D fingerprint proposed by Senese et al. [10]. In 2010, Rogers et al. [11] proposed molecular Morgan Fingerprints to study the neighborhood of each atom and the bonding connectivity between molecules. In 2020, Prasad and others [12] won the sixth round of the Statistical Assessment of the Modeling of Proteins and Ligands competition using the method of the Morgan fingerprint combined with deep learning, indicating that the Morgan fingerprint had good performance in the direction of prediction and screening.

The comparison in classification performance between various descriptors and molecular fingerprints is still a relatively ambiguous situation. The study by Mayr et al. [13] demonstrated that molecular fingerprint models contain a wider range of feature information than molecular descriptors obtained using convolutional models, while the experimental results by Wu et al. [14] showed the opposite. This discrepancy can be partly attributed to the dataset differences in evaluation metrics and molecular species. It is also partly due to the difference in the domains involved in designing the two types of molecular information. The study by Wu et al. [14] illustrated that molecular fingerprints are more specific to the chemical structure of molecules and the existence of some substructures. In contrast, molecular descriptors focus on the type and number of atoms in molecules and the shape of molecules.

In the previous study of Tseng et al. [15], an attempt was made to combine the two fingerprints, and, on this basis, a novel fingerprint was designed and showed a better predictive performance. Accordingly, Wang et al. [16] implemented joint fingerprinting and feature engineering. Traditional feature extraction methods include the genetic algorithm proposed by Pérez-Castillo et al. [17] and the partial least square method proposed by Su et al. [18]. The study by Hu et al. [19] showed that traditional feature extraction methods are cumbersome and need a wide range of professional knowledge, which significantly affects efficiency. Subsequent studies have shown that the proper selection and fusion of molecular fingerprints and molecular descriptors can significantly improve classification performance [20,21].

Although these models and methods have made some progress, many problems are still worthy of further exploration and research. One of the critical problems is the performance gap between different molecular descriptors and molecular fingerprints. There are some differences between molecular descriptors and molecular fingerprints, and it is necessary to make them more complementary. Another problem worth discussing is optimizing the structure of deep learning networks for feature extraction. With the development and advancement of deep learning, the effectiveness and efficiency of feature extraction are advancing continuously. Taherkhani’s study [22] found that, with deep learning algorithms, computers can automatically identify and filter out more important feature information, thus providing a more significant advantage in processing large-scale drug data. There is a significant difference between drug screening and traditional deep learning in that the number of labels in drug molecular datasets is uneven, sometimes even very different. This characteristic determines that overfitting is more likely to occur when using more complex network models for learning. The studies by Tetko et al. [23,24] also illustrated this problem.

Therefore, we focus on molecular fingerprints and molecular descriptors that contain more abundant molecular information. We combine both in a multimodal way to obtain complete information or features. Through the reasonable design of a deep learning network structure, we can achieve better feature extraction and avoid the occurrence of the overfitting phenomenon. This paper aims to achieve the above goals by designing the Molecular Information Fusion Neural Network (MIFNN). Two different patterns of molecular information are extracted from two different networks as feature information, and two parts of feature information are fused into the classification module to get the final classification results.

In MIFNN, we use two convolution networks with different dimensions to extract the characteristics of molecular information. In the one-dimensional convolution network, we process the molecular orientation information proposed by Yang et al. [7] and add an attention mechanism and bidirectional long short-term memory (bi-LSTM) to the one-dimensional convolution network. The LSTM module has been widely used in the natural language processing field. In the study of Xie [25], it was confirmed that a separate LSTM mechanism is conducive to screening drug molecules. However, the LSTM module often ignores the sequence information, and the molecular directed information expresses the molecules through the directional transmission between atoms. The research of Jiang et al. [26] and Chen et al. [27] showed that, if the sequence diagram information between atoms can be preserved completely, the subsequent feature extraction can be better carried out. Through the research of Lenselink et al. [28] and Öztürk et al. [29], we know that bidirectional LSTM can improve the effect of feature extraction of protein sequences under the framework of deep learning. Hence, we use bi-LSTM to avoid the disadvantages of the single LSTM mentioned above.

In the two-dimensional convolution network, the Morgan fingerprint is extracted. This is because there are many common digits in Morgan fingerprint; thus, the data can be spliced into a two-dimensional map to obtain complete feature information. For the classifier module, we use the particle swarm optimized support vector machine (PSO-SVM). This is the PSO algorithm optimization of the traditional SVM. The PSO algorithm is an adaptive algorithm based on alpha stable distribution and dynamic fractional calculus proposed by Deng [30]. After subsequent comparative experiments, in the research of Zhang et al. [31], the classifier optimized by the PSO algorithm was significantly improved.

Our MIFNN has two unique advantages: (1) feature extraction methods with different dimensions are used, and splicing and fusion methods are used to obtain more comprehensive molecular features; (2) the bi-LSTM mechanism and attention module are used for feature extraction to obtain complete molecular information. This can provide good support for subsequent prediction and classification, as well as achieve better results; (3) we choose the PSO support vector machine as the classifier, which can obtain more accurate classification results without easy overfitting [32]. We extensively evaluated our model and other recently released neural network structures for feature extraction and conducted several comparative experiments on eight publicly available test sets provided by Wu et al. [14] and Mayer et al. [13]. Our goal was to achieve more significant performance optimization than other network structures on the public dataset with the same evaluation index. According to our control experiment, our classification results had better performance in both the training set and the test set. For objectively evaluating the performance of the current model, a control group was established for different feature information using the classification network. The results show that there is still room for improvement in the structure of the deep learning model for subsequent optimization and improvement of the results.

## 2. Materials and Methods

### 2.1. Network Model

**Figure 1:**
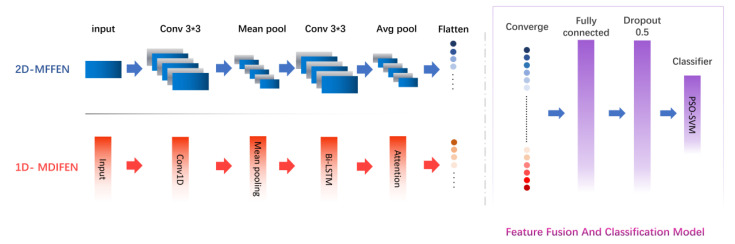
Molecular Information Fusion Neural Network model structure.

According to the characteristics of molecules, an MIFNN structure for molecular screening is proposed in this paper. The whole network structure can be divided into two parts. The first part is the molecular feature extraction module, which includes two subnetworks, namely, the Molecular Directed Information Feature Extraction Network (MDIFEN) and the Morgan Fingerprint Feature Extraction Network (MFFEN). MDIFEN can process the molecular directed information obtained through the transmission information network and enrich the information contained in the extracted features by the attention model. MFFEN is used to flatten the molecular Morgan fingerprint and send it to 2D-CNN for feature extraction. The second part is responsible for the fusion and classification of the features obtained by the molecular feature extraction module to achieve the purpose of molecular screening. The complete structure of the network model is shown in Figure 1. Next, we introduce these two parts in order.

#### 2.1.1. Molecular Feature Extraction Module based on bi-LSTM

The extraction and description of drug molecular information should be as comprehensive as possible and have wide applicability. To improve the performance of our network structure, we chose the molecular directed information and molecular Morgan fingerprint [11]. The specific acquisition and advantages of the above two kinds of molecular information are explained in detail in Section 2.2 and Section 2.3.

The molecular directed information based on the directed message passing neural network (D-MPNN) structure [7] places importance on the targeted transfer of information between molecules, which contains information on atomic species, molecular bond types, atomic neighborhoods, etc., on the basis of which Yang et al. achieved the task of screening out specific drugs in a large-scale drug molecule library [19]. Molecular Morgan fingerprinting has been combined with deep learning methods in recent studies to construct a method for predicting the high throughput of drugs and GraphDTA, a network structure for predicting drug–target affinity [12].

The whole feature extraction part of MIFNN consists of MDIFEN and MFFEN, where MDIFEN is used to extract 300 bit molecular directed information, while MFFEN is used to process 2048 bit molecular Morgan fingerprints.

**Figure 2:**
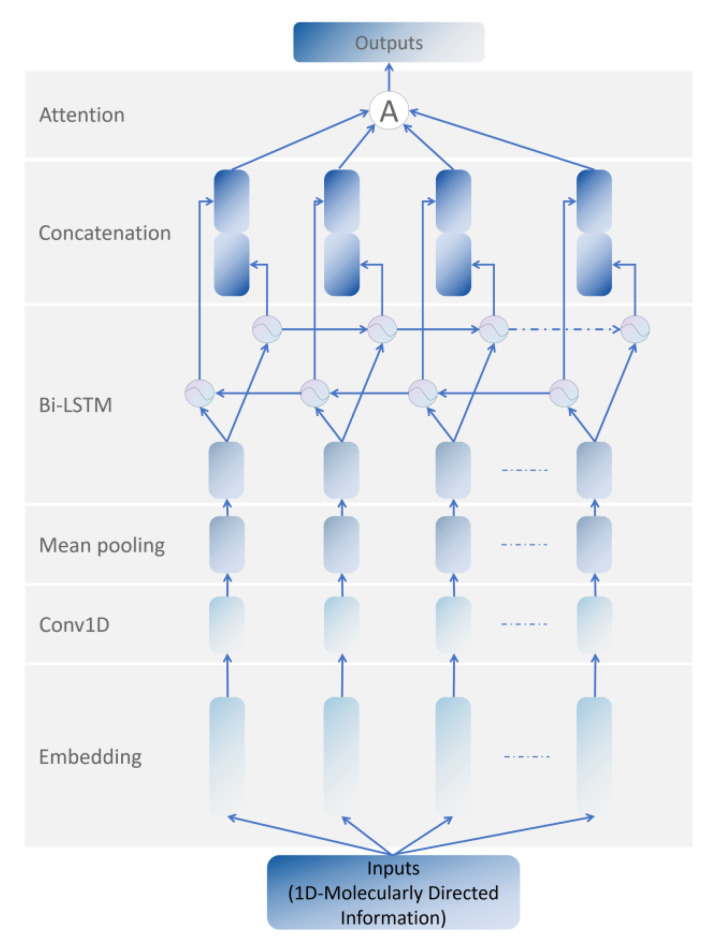
The 300 bit 1D Molecular Directed Information Feature Extraction Network.

MDIFEN includes the embedding layer, 1D convolution layer, mean pooling layer, bi-LSTM layer, concatenation layer, and attention module. We added a bi-LSTM layer to the traditional 1D-CNN to prevent gradient disappearance and gradient explosion. LSTM alone cannot deal with the sequential information flow well, and the molecular orientation information we use in MDIFEN has strict requirements for the order in the information sequence. Therefore, we use bi-LSTM, which has achieved good results in the NLP field, to optimize our structure. The extraction network of molecular directed information is shown in Figure 2.

**Figure 3:**
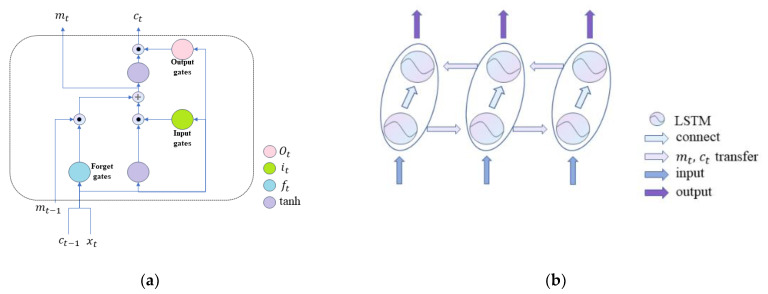
Structure of (**a**) LSTM and (**b**) Bi-LSTM.

LSTM is a variant of traditional RNN, and its specific structure is shown in Figure 3a. Bi-LSTM is a module including forward LSTM and backward LSTM. It can process the directional molecular information we use in the front and back directions to obtain more abundant features. The structure of bi-LSTM is shown in Figure 3b.

As shown in Figure 3a, the whole LSTM module includes the input gate $i_{t}$, output gate $o_{t}$, and forget gate $f_{t}$. The calculation process of gates is as follows:

(1) $$i_{t}=\sigma \left(W_{i}c_{t-1}+U_{i}x_{t}+\beta_{i}\right),$$

(2) $$\;{\tilde{m}}_{t}=tanh\left(W_{m}c_{t-1}+U_{m}x_{t}+\beta_{m}\right),$$

(3) $$m_{t}=f_{t}⊙m_{t-1}+i_{t}⊙{\tilde{m}}_{t}\;\left(a\right),$$

(4) $$o_{t}=\sigma \left(W_{o}c_{t-1}+U_{o}x_{t}+\beta_{o}\right),$$

(5) $$c_{t}=o_{t}⊙tanh\left(m_{t}\right),$$

(6) $$f_{t}=\sigma \left(W_{f}c_{t-1}+U_{f}x_{t}+\beta_{f}\right),$$

where W and U represent the offset matrix, β Indicates bias, and $m_{t}$ indicates a memory cell of LSTM. $x_{t}$ and $c_{t}$ are the input vector and the hidden state vector at time t, respectively.

According to the previous statement, the bi-LSTM structure is used, with bidirectional hidden information forward ${\vec{c}}_{t}$ and backward ${\overset{\leftarrow }{c}}_{t}$, to enable LSTM to completely obtain the context information. $LSTM\left(\cdot \right)$ represents the operation process of LSTM module. The update of the two hidden pieces of information and the calculation process of the subsequent concatenation layer are as follows:

(7) $${\vec{c}}_{t_{LSTM}}=\vec{LSTM}\left(m_{t}\right),t\in \left[1,n\right],$$

(8) $${\overset{\leftarrow }{c}}_{t_{LSTM}}=\overset{\leftarrow }{LSTM}\left(m_{t}\right),t\in \left[n,1\right],$$

(9) $$c_{t_{LSTM}}=\left[{\vec{c}}_{t_{LSTM}},{\overset{\leftarrow }{c}}_{t_{LSTM}}\right].$$

After bi-LSTM is processed, it is sent to the attention module for the final processing of feature extraction. The calculation process of this module is as follows:

(10) $$\eta_{t_{LSTM}}=tanh\left(W_{w_{LSTM}}h_{t_{LSTM}}+\beta_{w_{LSTM}}\right),$$

(11) $$\alpha_{t_{LSTM}}=\frac{\exp\left(\eta^{𝞣}{}_{t_{LSTM}}\eta_{w_{LSTM}}\right)}{\sum_{t}\exp\left(\eta^{𝞣}{}_{t_{LSTM}}\eta_{w_{LSTM}}\right)\;},$$

(12) $$s_{LSTM}=\sum_{t}\alpha_{t_{LSTM}}c_{t_{LSTM}},$$

where $\eta_{t}$ is the hidden representation of $c_{t}$, $\eta_{w}$ is the context vector that is constantly initialized in the training stage, the importance of a part of the molecular information is calculated through the similarity between $\eta_{t}$ and $\eta_{w}$, and the finally obtained weighted feature information $s_{LSTM}$ is sent to the subsequent fusion and classification module for processing.

**Figure 4:**
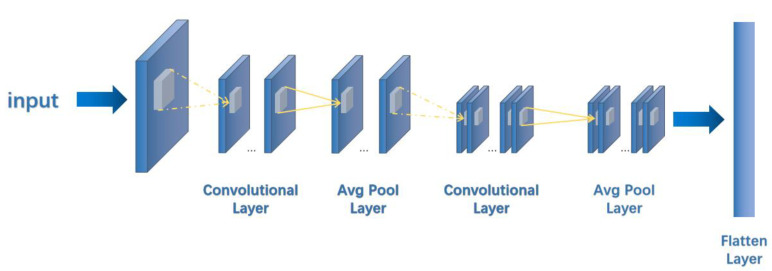
The 2048 bit 2D Morgan Fingerprint Feature Extraction Network. The dashed line shows the convolution process, and the solid line shows the pooling process.

In MFFEN, the 2048 bit molecular Morgan fingerprint is first converted into a 32 × 64 matrix as input, and 2D convolution is used for convolution calculation throughout the process. The pooling layer is also used for average pooling, and the padding is all ‘SAME’. Throughout the model network operation, the learning rate is set to 0.001, the Adam optimizer is used, the number of iterations is 100, and the dropout is 0.5 for training and 1.0 for testing. The network structure of the Morgan fingerprint feature extraction network is shown in Figure 4.

Before features of different dimensions are fused, the data need to be flattened to one dimension. The flattening operation can be expressed as

(13) $$P_{i}=\left(\begin{array}{ccc} a_{11} & \ldots & a_{1n}\\ \vdots & \ddots & \vdots \\ a_{m1} & \cdots & a_{mn} \end{array}\right),$$

(14) $$p_{i}^{T}=flatten\left(P_{i}\right)=\left[a_{11},\ldots ,a_{1n},a_{21},\ldots ,a_{2n},\ldots ,a_{m1},\ldots a_{mn}\right],$$

where $P_{i}$ is a m×n feature map, a represents the feature values, and $p_{i}$ represents the 1D vector after data spreading.

#### 2.1.2. Feature Fusion and Classification Model

In this module, a fusion layer is adopted first to connect the previously extracted features containing different information [33]. When the features are completely spliced, they are sent to the full connection layer and the discard layer. Accordingly, we can obtain better classification results in the follow-up. Lastly, these features are sent to PSO-SVM for classification.

The adopted fusion strategy is feature fusion, a mid-level fusion between data fusion and decision fusion. It avoids both a large amount of computation and a large amount of information detail loss. The fusion is performed by using a concatenated fusion, where the feature vectors from the previous 1D-CNN and 2D-CNN spread are concatenated. The fusion equation is

(15) $$f_{i}^{T}=FF\left(P_{i1},P_{i2}\right)=\left[a_{111},\ldots ,a_{nn1},a_{112},\ldots ,a_{nn2}\right],$$

where $P_{i1}$, $P_{i2}$ are 1D eigenvectors and 2D eigenvectors, respectively, and a represents a single eigenvalue in two different vectors.

With the help of the flattening and feature fusion strategy, the model can obtain more comprehensive and accurate evaluation results. The fused vectors are fed into the FC layer.

We add an FC layer after the feature fusion layer to integrate various features extracted from different convolutional kernels in the previous layers to achieve the final classification. The implementation of the FC layer is

(16) $$a^{m}=f\left(x^{m}\right)=f\left(\omega^{m}a^{m-1}+b^{m}\right).$$

Each feature value $a^{m}$ after fusion processing is calculated with a weight $\omega^{m}$ and a bias $b^{m}$. The new feature values are obtained after the activation function, which can achieve better classification results.

We use the dropout layer to improve the generalization ability and robustness of the model. The dropout parameter set here is 0.5. Its output is used as the final extracted feature and transmitted to the classification layer.

SVM is a sparse and robust classifier that uses the hinge loss function to compute the empirical risk and adds a regularization term to the solution system to optimize the structural risk [32]. It is one of the common kernel learning methods. We choose SVM as the classifier in this paper because overfitting tends to occur when using complex machine learning modules for similar tasks classification [34].

At the same time, SVM as a traditional machine learning classifier has been improved continuously in recent years, with PSO-SVM representing one of the SVM variants. The PSO algorithm is used to optimize the kernel parameter g and penalty term C in SVM, which can improve the generalization ability of the whole SVM classifier and obtain better results. The calculation process of PSO-SVM is as follows:

(17) $$\;Fit\left(x\right)=\frac{1}{n}\overset{\underset{n}{i=1}}{\sum \;}{\left(f_{i}\left(x\right)-y_{i}\left(x\right)\right)}^{2},$$

where $Fit\left(x\right)$ is the fitness function of the whole optimization algorithm. A larger value denotes a better optimization effect. $f_{i}\left(x\right)$ and $y_{i}\left(x\right)$ represent the real label and the prediction label, respectively, and n is the number of samples. The abovementioned C and g are combined as PSO particles, which can be abstracted as a set of points in the plane. The particle’s position L and moving speed v are updated by

(18) $$\;v_{id}^{k}=U_{i}v_{id}^{k-1}+c_{1}rand_{1}\left(P_{best_{i}}-L_{id}^{k-1}\right)+c_{2}rand_{2}\left(G_{best_{i}}-L_{id}^{k-1}\right),$$

(19) $$\;L_{id}^{k}=L_{id}^{k-1}+v_{id}^{k},$$

where U is the inertia weight, which is used to control the speed of overall optimization, c is the learning factor, rand is an independent random number in [0,1], d is the dimension of the solution vector, i is the number of particles that may be composed of all C and g, and $G_{\mathrm{best}}$ and $p_{\mathrm{best}}$ are the global optimal solution and the partial optimal solution, respectively. The optimized parameters are sent to SVM for final classification. The calculation process of classification is as follows:

(20) $$\;Y\left(f\right)=sgn(g\left(f\right))=sgn\left(\overset{\underset{N}{i=1}}{\sum \;}a_{i}^{*}y_{i}\kappa \left(f_{i},f\right)+b^{*}\right),f\in R_{N},$$

(21) $$\;\kappa \left(f_{i},f\right)=exp\left(-\frac{{\left\|f_{i}-f\right\|}^{2}}{2\sigma^{2}}\right),$$

where f represents the eigenvector. Gaussian kernel functions κ and σ are used because this SVM classification is in high-dimensional space, where σ is the width parameter of the kernel function.

It is worth mentioning that, when using SVM as the classification layer to perform the final classification, the result obtained is the distance from the experimental sample to the hyperplane, which is the basis for SVM classification [35]. However, in this research, we expect the classification result to be the probability of the effectiveness of a drug molecule for given disease; therefore, we need to process the output of SVM. We consider the transformation of the SVM output into posterior probabilities using the sigmoid-fitting method, and the steps and formulas for processing are as follows:

(22) $$P(y=1|Y)=\frac{1}{1+exp(Af+B)},$$

where A and B are the parameters to be fitted, and Y is the output of the sample. The advantage of the sigmoid-fitting method is that it can estimate the posterior probabilities well while maintaining the sparsity of the SVM. This is implemented through the Libsvm library function in Matlab to complete the program [36].

### 2.2. Directed Message Passing Information

The message passing neural network (MPNN) is a network structure that operates on graph G [37]. It uses the atomic feature $a_{p}$ with the bond feature $b_{p}$ to represent the information of the molecule. The network structure operates in two phases: the generation of molecular representations phase and the reading phase. In the first stage, a symbol representing the molecular features is generated by processing the transfer information in the molecular graph. In the second stage, valuable molecular characteristics are predicted on the basis of this symbol. The formulas for the generation of molecular representations are

(23) $$i_{p}^{s+1}=\underset{q\in N\left(p\right)}{\sum }M_{S}\left(c_{p}^{s},c_{q}^{s},b_{pq}\right),$$

(24) $$c_{p}^{s+1}=U_{S}\left(c_{p}^{s},i_{p}^{s+1}\right).$$

There are s steps in the first stage, where a certain atom p, hidden state $c_{p}^{s}$, and message $i_{p}^{s}$ are updated. $M_{S}$ and $U_{S}$ in the above equation are the message function and the atomic fixed-point update function, respectively. In addition, $N\left(p\right)$ is a set of neighboring atoms in graph G.

**Figure 5:**
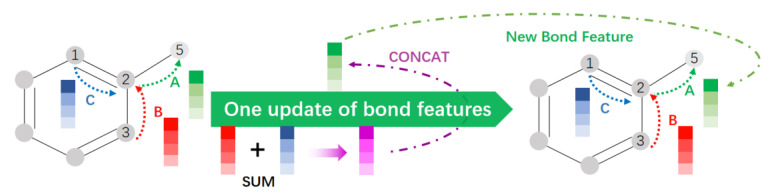
Update of key feature information in D-MPNN.

The D-MPNN is a new network structure that improves the MPNN described above [38]. This network structure uses information related to directed bonds instead of information related to atoms, which can avoid the redundancy that arises when atomic information is passed undirected in the graph [39]. As shown in Figure 5, after numbering the atom information and bond information, bond C and bond B transmit bond characteristic information to atom 2 and perform summation of the corresponding bits. This is then linked with the information about bond A itself from atom 2 to atom 5 to update the information about the bond A features. Such an update step can avoid the generation of redundant information, while enabling concise feature information and improved efficiency in subsequent feature processing.

Unlike the formula mentioned by MPNN, directed hidden states $c_{pq}^{s}$ and directed messages $i_{pq}^{s}$ instead of $c_{p}^{s}$ and $i_{p}^{s}$ are used in D-MPNN. Because of the existence of vector-like directionality, messages $i_{pq}$ from p to q and messages $i_{qp}$ from q to p are completely different for atoms, but similar for hidden messages $c_{pq}$ and $c_{qp}$. The D-MPNN principle equations are as follows [7]:

(25) $$i_{pq}^{s+1}=\underset{j\in \left\{N\left(p\right)\backslash q\right\}}{\sum }M_{s}\left(a_{p},a_{q},c_{jp}^{s}\right),$$

(26) $$c_{pq}^{s+1}=U_{s}\left(c_{pq}^{s},i_{pq}^{s+1}\right).$$

It is worth mentioning that $i_{pq}^{s+1}$ is a relatively independent state that does not have much correlation with $i_{pq}^{s}$. Before each message passing operation, the hidden messages are initialized, using the following initialization formula [40]:

(27) $$c_{pq}^{0}=\tau \left(T_{l}cat\left(a_{p},b_{pq}\right)\right),$$

where $T_{l}\in R^{h*h_{l}}$ is a learning matrix, $cat\left(a_{p},b_{pq}\right)\in R^{h_{l}}$ is a connection between atomic features $a_{p}$ and bond features $b_{pq}$, and τ is the Relu activation function.

We chose the message function $M_{s}$ with the update function $U_{S}$ defined as

(28) $$M_{s}\left(a_{p},a_{q},c_{pq}^{s}\right)=c_{pq}^{s},$$

(29) $$U_{s}\left(c_{pq}^{s},i_{pq}^{s+1}\right)=U\left(c_{pq}^{s},i_{pq}^{s+1}\right)=\tau \left(c_{pq}^{0}+T_{i}i_{pq}^{s+1}\right).$$

Note that the addition of $c_{pq}^{0}$ at every step provides a skip connection to the original feature vector for that edge. Lastly, we return to the atomic representation of the molecule by summing the characteristics of the incoming bonds according to

(30) $$i_{p}=\underset{j\in N\left(p\right)}{\sum }c_{jp}^{T},$$

(31) $$c_{p}=\tau \left(T_{a}cat\left(a_{p},i_{p}\right)\right).$$

According to the process mentioned above, the messaging phases of D-MPNN can be summarized in the following order:

(32) $$c_{pq}^{0}=\tau (T_{i}cat\left(a_{p},b_{pq}\right)),$$

(33) $$i_{pq}^{s+1}=\underset{j\in N\left(p\right)\backslash q}{\sum }c_{jp}^{s}.$$

For $s\in \left\{1,\ldots ,T\right\}$,

(34) $$c_{pq}^{s+1}=\tau \left(c_{pq}^{0}+T_{i}i_{pq}^{s+1}\right),$$

(35) $$i_{p}=\underset{q\in N\left(p\right)}{\sum }c_{pq}^{T}.$$

The molecular directed passing information we need is obtained at this point by summing the hidden information. This information is further processed in the subsequent network to extract features and contribute to the subsequent classification [7].

(36) $$DPI=\underset{p\in G}{\sum }c_{p}.$$

### 2.3. Morgan Fingerprint

Morgan fingerprints, which are circular and topological fingerprints, are obtained by adapting the standard Morgan algorithm [41]. They can be roughly equated to extended-connectivity fingerprints (ECFPs).

These fingerprints bring some advantages: (1) fast computation, (2) no need for predefinition (can represent an infinite number of different molecular features), (3) contain chiral information, with each element in the fingerprint representing a specific substructure, (4) can be easily analyzed and interpreted, (5) can be modified accordingly to different needs, etc. These fingerprints are originally designed to search for molecular features related to activity rather than substructure searches [42]. They can also be used in the direction of similarity search, clustering, virtual screening, etc. The fingerprint generation process is broadly divided into the following steps:

Step.1. Atom initialization. Assign an integer identifier to each atom.

Step.2. Iterative update. Take each heavy atom as the center and merge it in the surrounding circle of heavy atoms until the specified radius is reached.

Step.3. Feature generation. Perform operations on substructures and generate a list of features.

In contrast to ECFPs, which capture precise substructure details, functional class fingerprints (FCFPs) are more generalized, allowing the same class of functional groups to be used as a feature structure. Both features can be implemented in RDKit using ‘GetMorganFingerprint’. In this research, the RDKit package in Python is used for the extraction of binary molecular Morgan fingerprints [43].

**Figure 6:**
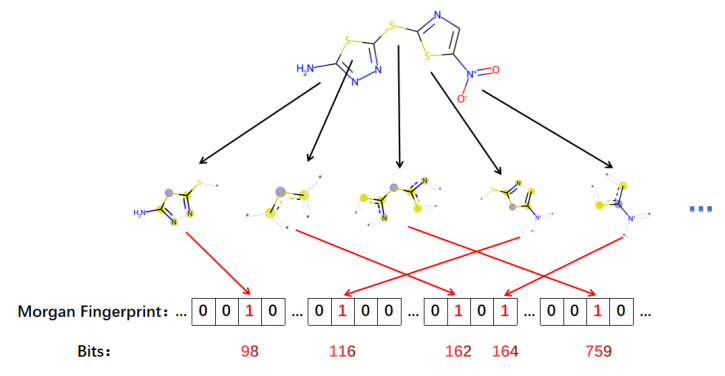
Schematic diagram of Morgan fingerprint generation.

We set the number of bits of the desired molecular fingerprint to 2048 before running the code. When obtaining the corresponding fingerprint, we transformed it from one-dimensional information (2048 × 1) to two-dimensional information (32 × 64) to facilitate further extraction and classification of features. The specific acquisition steps are illustrated in Figure 6.

## 3. Experiment

In this section, we conduct extensive comparative experimental studies on the proposed MIFNN model to validate the method’s effectiveness. The CPU of the device we use is Intel (R)_ Core (TM)_ 9-10920x, the graphics card is gtx3080, and the system is Ubuntu 16.04.

### 3.1. Model Experiment

#### 3.1.1. Data

The data used in this paper are filtered according to publicly available datasets that are more used in the field and contain thousands to more than 100,000 drug molecules with different structures. Detailed statistical information on all datasets is listed in Table 1. In some of these datasets, the classification labels are incomplete. Therefore, the original labels are sorted to obtain the valid and invalid labels needed for dichotomization. In all datasets, some molecular drugs that do not represent the complete vector information and molecular fingerprints are intentionally ignored.

#### 3.1.2. Model Performance Evaluation

It is worth mentioning that, because the amount of data expressed as valid in these datasets is small, and two types of labeled data appear extremely unbalanced as a dichotomy, the receiver operating characteristic (ROC) curve is used to judge the network performance. The ROC is a curve based on a series of different dichotomies (cutoff value or decision threshold), with the true positive rate as the vertical coordinate and the false positive rate as the horizontal coordinate. The AUC (area under curve) is defined as the area under the ROC curve. We use the AUC value as the evaluation standard of the model. When the AUC value is larger, the classification effect is better. The calculation formulas of the horizontal and vertical coordinates of the ROC curve are as follows:

(37) $$\mathrm{TPrate}=\frac{\mathrm{TP}}{\mathrm{TP}+\mathrm{FN}},$$

(38) $$\mathrm{FPrate}=\frac{\mathrm{FP}}{\mathrm{FP}+\mathrm{TN}},$$

where TP, TN, FP, and FN respectively represent true positives, true negatives, false positives, and false negatives, respectively.

### 3.2. Experimental Results

To verify the performance of our MIFNN, we set up two control experiments in different directions. The first was to compare the complete MIFNN model with other baseline models with good results in this field. The second was to simplify MIFNN to verify the optimization effect of the modified algorithm and network structure.

#### 3.2.1. Improvements to Other Baseline Models

**Figure 7:**
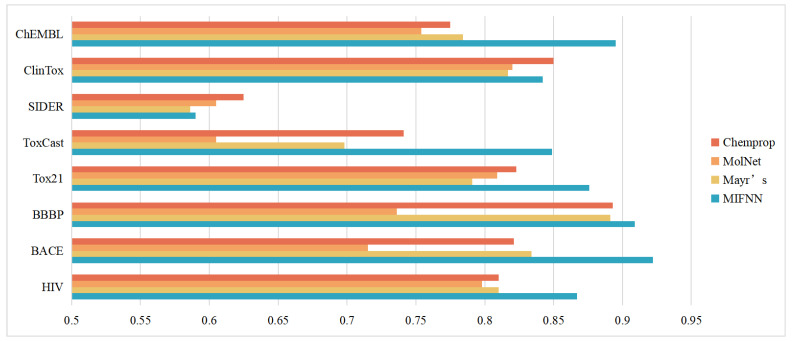
Comparison results with baseline models.

We first compared our model with several different baseline models. The baseline models used in this section included the model mentioned in MoleculeNet, the model Chemprop proposed by Yang et al., and the model proposed by Mayer et al. It is worth mentioning that, to make the verification results more convincing, we chose the model that performed best against the same dataset in MoleculeNet. The specific comparison results are shown in Table 2 and Figure 7. From Figure 7, we can see that our MIFNN model significantly improved the classification effect of datasets except for SIDER. At the same time, the classification effect of the Chemprop model was second only to our MIFNN in datasets other than ChEMBL. The performance optimization of HIV, BACE, Tox21, and ToxCast was more obvious than that of other baseline models. In these four datasets, the AUC of MIFNN increased by 7.0%, 11.6%, 6.4%, and 14.6%, respectively, compared with the optimal solution in the baseline model.

The models in MolNet had different optimal solutions for different datasets. However, they had one thing in common with other baseline models, i.e., the use of a single dimension of molecular information. Our MIFNN uses two different dimensions of molecular information for feature extraction. This led to our classification effect outperforming the baseline models. Our MIFNN model had the best performance in all datasets except SIDER.

#### 3.2.2. Improvement under Different Conditions

**Figure 8:**
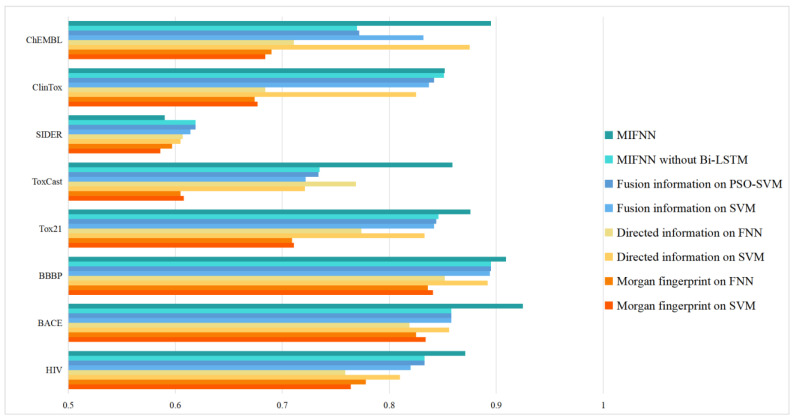
Model optimization results.

**Figure 9:**
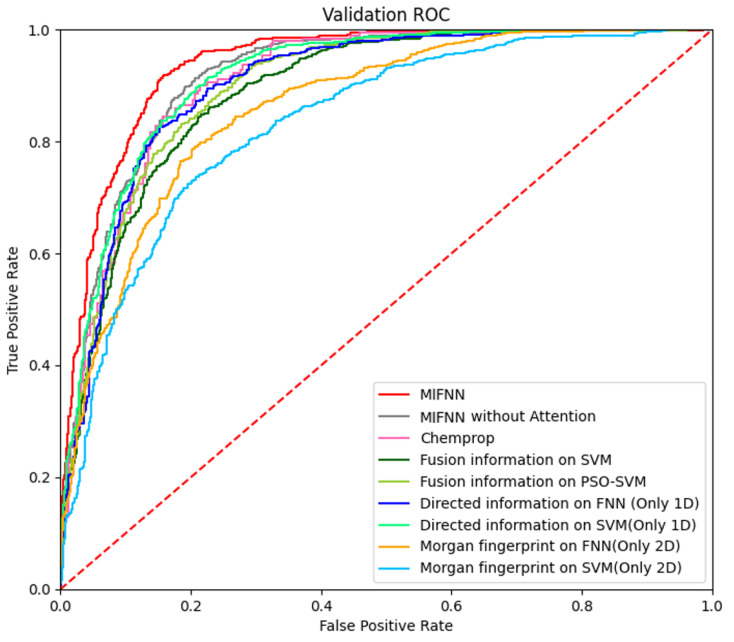
Visualization of model optimization results.

As mentioned above, MIFNN is composed of various modules. To verify the functions of various modules, we set up control experiments. The performance of the complete MIFNN was verified by simplifying the model. The simplified model included (1) using a convolution network of a single dimension in the feature extraction part of molecular information and using a simple classifier model such as FFN for comparison, (2) removing the bidirectional LSTM layer and attention module from the complete MIFNN model, and (3) comparing the traditional SVM with PSO-SVM. The specific comparison results are shown in Table 3. The visualization of the experimental results is shown in Figure 8. In order to more intuitively see the optimization brought about by the model improvement, we selected the BACE dataset to visualize the ROC curve. The adopted structure is provided in Table 3. In addition to our simplified model, we added the Chemprop model with relatively good performance in Figure 7 to obtain the ROC curve. The specific ROC curve is shown in Figure 9.

We conducted a series of comparative experiments based on the D-MPNN network in the citation. By comparing the experimental results in the citation and the specific experimental results of our MIFNN, we can see that the AUC values obtained from our network were improved by 5.6%, 6.6%, 3.5%, 16.9%, and 6.4% for HIV, BACE, Tox21, ToxCast, and ChEMBL. In general, the optimization of the model can be considered more obvious when the AUC value increased by 0.03 or more.

In the ClinTox and BBBP datasets, the AUC values obtained by our network were 0.1%, and 1.6% higher than the highest values of other models, respectively. This kind of situation can be considered a slight optimization, but the improvement effect was not obvious. Lastly, on SIDER, our model did not work as well as other traditional models.

By observing Table 3, we can know that, after the feature information fusion of different dimensions, we could obtain a better classification effect. This was often the reason why our model performed better when compared with the baseline model. Using PSO-SVM for classification, the effect was also improved, because the PSO algorithm optimized and integrated various parameters in SVM. The bi-LSTM module could obtain more abundant molecular information, make full preparations for subsequent feature extraction, and improve the classification effect.

In general, in the eight datasets, we performed a complete comparison with the current common baseline models with good results. Moreover, a comparative experiment was performed for the optimization points of the model to verify that our optimization method was effective. In the four datasets, MIFNN was much better than the best baseline model, while it was slightly better than the baseline model in three datasets, and only slightly lower than the Chemprop model in one dataset (i.e., SIDER); however, the difference was very small. This shows that MIFNN is better than the best baseline model. A more representative advantage is that the MIFNN model structure of each dataset was roughly the same, whereas, in the previous methods, the optimal model of different datasets was different. This shows that our method is more universal and can better complete the screening task of different drug molecular datasets.

## 4. Conclusions

This paper presented a relatively detailed comparison of molecular property prediction models based on fixed descriptors, molecular fingerprints, and our proposed fusion features through multiple experiments on eight public datasets. Table 2 shows a comparison of our proposed model, MIFNN, with each baseline model. Our model achieved better performance on seven out of eight public datasets: HIV, BBBP, PCBA, BACE, Tox21, ToxCast, and ClinTox. In all eight datasets, no single model performance was consistently the highest.

Our future work will constitute two directions. The first is to try to add a residual module or a self-attention mechanism module to the classification model to obtain more accurate classification results. The continuous development of deep learning will provide us with the optimal structure to optimize our network and make it more comfortable with unbalanced datasets. The second is how to expand the training set by discovering new molecular descriptors or generating virtual fingerprint information for the problem of the small number of datasets during training.

## Figures and Tables

**Table 1:** Details of the dataset used.

Data Set	Category	Description	Size
HIV	Biophysics	Inhibition of HIV replication	41,127
BACE	Biophysics	Inhibition of human β-secretase 1	1513
BBBP	Physiology	Ability to penetrate the blood–brain barrier	2039
Tox21	Physiology	Toxicity	7831
ToxCast	Physiology	Toxicity	8576
SIDER	Physiology	Side-effects of drugs	1427
ClinTox	Physiology	Toxicity	1478
ChEMBL	Physiology	Biological assays	456,331

All datasets were judged using the ROC-AUC.

**Table 2:** Comparison of AUC values for MIFNN and baseline models (underlined values are maximum values; bolded values are significantly higher than those of other models).

Dataset	MIFNN	Mayr’s	MolNet	Chemprop
HIV	**0.867**	0.81	0.798	0.81
BACE	**0.922**	0.834	0.715	0.821
BBBP	0.909	0.891	0.736	0.893
Tox21	**0.876**	0.791	0.809	0.823
ToxCast	**0.849**	0.698	0.605	0.741
SIDER	0.59	0.586	0.605	0.625
ClinTox	0.842	0.817	0.82	0.85
ChEMBL	0.895	0.784	0.754	0.775

**Table 3:** Comparison of AUC values for various models. (underlined values are maximum values; bolded values are significantly higher than those of other models).

Dataset	Morgan Fingerprint on SVM	Morgan Fingerprint on FNN	Directed Information on SVM	Directed Information on FNN	Fusion Information on SVM	Fusion Information on PSO-SVM	MIFNN without Bi-LSTM	MIFNN
HIV	0.764	0.778	0.81	0.759	0.811	0.816	0.833	**0.871**
BACE	0.834	0.825	0.856	0.819	0.837	0.862	0.868	**0.925**
BBBP	0.841	0.836	0.89	0.852	0.870	0.877	0.895	0.909
Tox21	0.711	0.709	0.833	0.774	0.817	0.839	0.846	**0.876**
ToxCast	0.608	0.605	0.721	0.769	0.724	0.731	0.735	**0.859**
SIDER	0.586	0.597	0.605	0.607	0.601	0.615	0.619	0.59
ClinTox	0.677	0.674	0.825	0.684	0.696	0.815	0.851	0.852
ChEMBL	0.684	0.69	0.875	0.711	0.841	0.786	0.77	**0.895**

## Data Availability

All data, models, and code generated or used during the study appear in the submitted article.
